# A new Echimyidae (Mammalia, Rodentia) host of *Amblyomma ovale* Koch, 1844 in the eastern Brazilian Amazon, and a pathogen survey

**DOI:** 10.1590/S1984-29612025044

**Published:** 2025-08-07

**Authors:** Darlison Chagas-de-Souza, Cláudia Regina Silva, Tássio Alves-Coêlho, Ricardo Bassini-Silva, Fernando de Castro Jacinavicius, Marcelo Bahia Labruna, Lúcio André Viana

**Affiliations:** 1 Programa de Pós-graduação em Biodiversidade Tropical, Universidade Federal do Amapá – UNIFAP, Macapá, AP, Brasil; 2 Laboratório de Estudos Morfofisiológicos e Parasitários, Departamento de Ciências Biológicas e da Saúde, Universidade Federal do Amapá – UNIFAP, Macapá, AP, Brasil; 3 Laboratório de Mastozoologia, Instituto de Pesquisas Científicas e Tecnológicas do Estado do Amapá – IEPA, Macapá, AP, Brasil; 4 Laboratório de Coleções Zoológicas, Instituto Butantan, São Paulo, SP, Brasil; 5 Departamento de Biologia Animal, Instituto de Biologia, Universidade Estadual de Campinas – UNICAMP, Campinas, SP, Brasil; 6 Departamento de Medicina Veterinária Preventiva e Saúde Animal, Faculdade de Medicina Veterinária e Zootecnia – FMVZ, Universidade de São Paulo – USP, São Paulo, SP, Brasil

**Keywords:** Amblyomma, Brazilian Amazon, host-parasite interactions, Rodentia, Ixodidae, Amblyomma, Amazônia brasileira, interações hospedeiro-parasita, Rodentia, Ixodidae

## Abstract

The genus *Amblyomma* has the largest number of tick species in the Neotropical region. *Amblyomma ovale* displays high ecological plasticity and can be found in different habitats and a variety of vertebrate hosts. Small mammals of the family Echimyidae have been identified as hosts for this species. However, studies investigating their role as hosts of these ticks in the Amazon region are lacking. The present study aims to record the parasitic association between *A. ovale* and a Echimyidae species (*Proechimys cuvieri*) in the extreme north of the Brazilian Amazon, providing information about the research of potentially pathogenic organisms. Eight nymphs were collected and subjected to taxonomic and molecular identification, and pathogen screening was performed on the family Anaplasmataceae agents and the genera *Bartonella*, *Coxiella*, *Hepatozoon*, *Mycoplasma*, and *Rickettsia.* All the ticks were identified morphologically and molecularly as *A. ovale*, but PCR assays for pathogen detection showed no positive results for the target genes. This is the first time that *A. ovale* has been associated with *P. cuvieri.*

## Resumo

O gênero *Amblyomma* possui o maior número de espécies de carrapatos na região Neotropical. *Amblyomma ovale* apresenta alta plasticidade ecológica e pode ser encontrado em diferentes habitats e em inúmeros hospedeiros vertebrados. Pequenos mamíferos da família Echimyidae foram identificados como hospedeiros para esta espécie. No entanto, estudos que investigam seu papel como hospedeiros desses carrapatos na região amazônica são inexistentes. O presente estudo tem como objetivo registrar a associação parasitária entre *A. ovale* e uma espécie de Echimyidae (*Proechimys cuvieri)* no extremo norte da Amazônia brasileira, fornecendo informações sobre a pesquisa de organismos potencialmente patogênicos. Oito ninfas de carrapatos foram coletadas e submetidas à identificação taxonômica e molecular, e a triagem de patógenos foi realizada tendo como alvo agentes da família Anaplasmataceae e nos gêneros *Bartonella, Coxiella, Hepatozoon, Mycoplasma* e *Rickettsia*. Todos os carrapatos foram identificados morfológica e molecularmente como *A. ovale*, no entanto os ensaios de PCR para detecção de patógenos não mostraram resultados positivos para os genes-alvo. Esta é a primeira vez que *A. ovale* foi associado a *P. cuvieri*.

## Introduction

The genus *Amblyomma* Koch, 1844 comprises approximately 140 tick species worldwide, corresponding to approximately 19% of the diversity of known Ixodidae. The largest number of representatives of this genus were established in the Gondwana region and the Neotropics. This is the most speciose group in the Southern Cone, comprising 26 recognized species (Soares et al., 2023).

Among the hard ticks of the genus *Amblyomma*, *A. ovale* Koch, 1844, is a Neotropical tick with high ecological plasticity found in areas with different climates and vegetation characteristics (Guglielmone et al., 2021). The distribution of this species has been confirmed in Argentina, Belize, Bolivia, Brazil, Colombia, Costa Rica, Ecuador, El Salvador, French Guiana, Guatemala, Guyana, Mexico, Nicaragua, Panama, Paraguay, Peru, Suriname, Trinidad and Tobago and Venezuela, with sporadic records in Nearctic localities within Mexico and the United States (Guglielmone et al., 2021).

*Amblyomma ovale* is a three-host tick, whose adult stage parasitizes mostly mammals of the order Carnivora, while its immature stages parasitize rodents and other small mammals (Sabatini et al., 2010). The major veterinary importance of this tick in Latin America stems from the fact that it is one of the most common ticks that infest domestic dogs in rural areas (Sabatini et al., 2010), where it is a vector of *Hepatozoon canis* (Forlano et al., 2005). *Amblyomma ovale* is one of the most common human-biting ticks in Brazil (Nogueira et al., 2022), where it is the main vector of *Rickettsia parkeri* strain Atlantic rainforest, an emerging agent of spotted fever disease in South America (Silva-Ramos et al., 2021).

Small mammals of the family Echimyidae have been identified as hosts of the tick *A. ovale*. However, the specific role of these mammals as hosts of these ticks in the Amazon region, particularly in the context of potential pathogen infection, remains largely unexplored. Known as spiny rats, the genus *Proechimys* is among the most abundant terrestrial mammals in Amazonian forests (Silva et al., 2018). The ‘cuvieri group’ comprises only one nominal form, *Proechimys cuvieri* Petter 1978, distributed throughout the Amazon basin from eastern Ecuador and Peru, and the states of Acre to Amapá in Brazil, to the Guianas and Venezuela (Patton et al., 2015). In this study we recorded the parasitic association between *A. ovale* and an Echimyidae in the extreme northern Brazilian Amazon, providing potentially valuable insights into the presence of pathogenic organisms.

## Material and Methods

**Figure 1:**
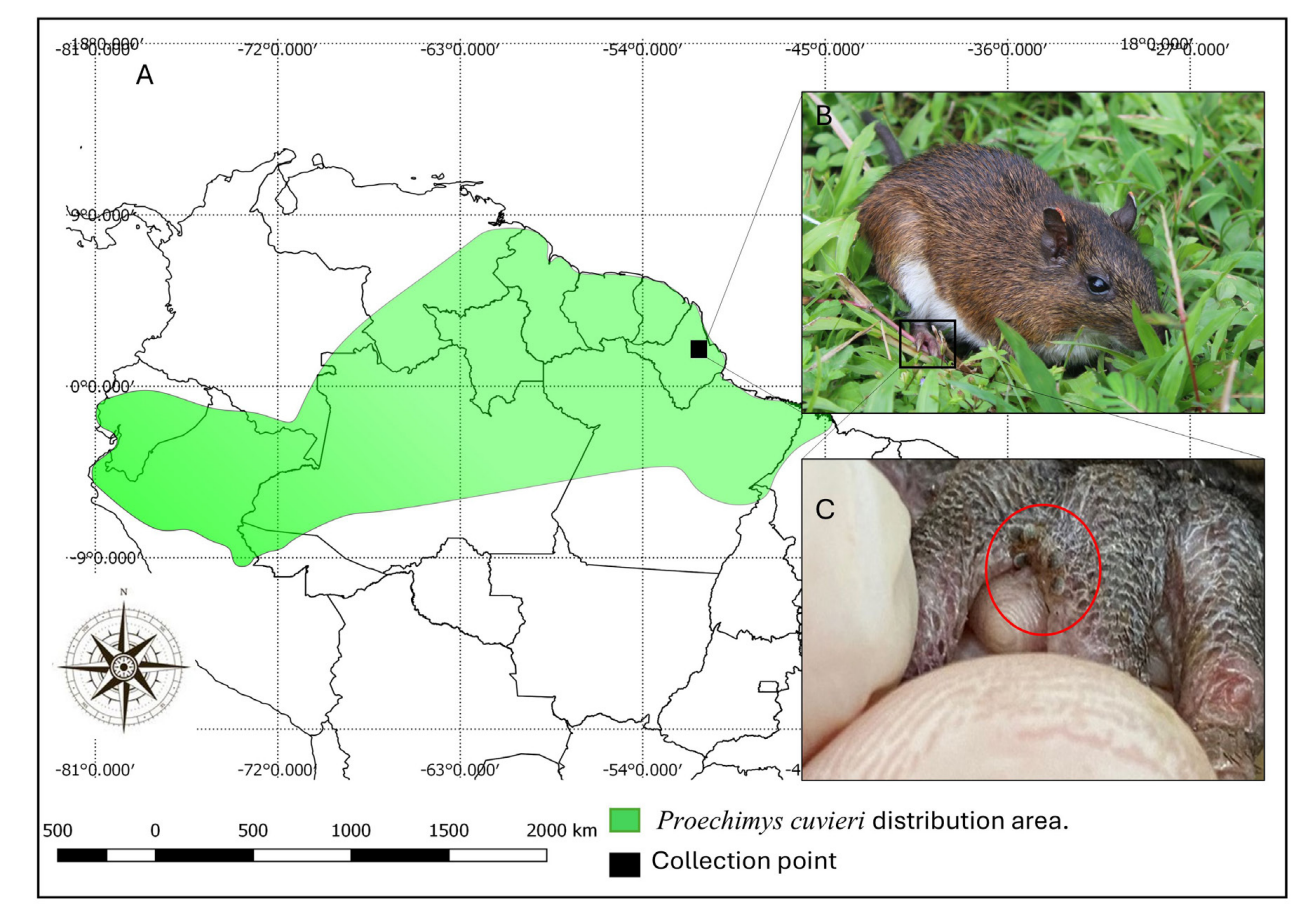
(A) Map with the distribution and locality of the *Proechimys cuvieri* collection; (B) Specimen of *P. cuvieri*; (C) Visualization of ticks found on the host.

During the taxidermy process at the Mammal Laboratory (LAMAM) of the Institute for Scientific and Technological Research of the State of Amapá (IEPA), two specimens of Cuvier's spiny rat, *Proechimys cuvieri* Petter, 1978, from a floodplain forest on the Amazon River in the municipality of Itaubal, state of Amapá, Brazil (0°36'9.43”N / 50°41'59.73”W) were subjected to ectoparasite research (Figure 1). The taxonomic classification of the hosts was determined based on morphological and morphometric characteristics. This determination was made using a Proechimys cuvieri voucher series, which underwent morphological and molecular identification by Silva et al. (2018). These specimens are currently stored in the mammal collection of the Scientific Collection Fauna of Amapá (CCFA), which is located at the Instituto de Pesquisas Científicas e Tecnológicas do Estado do Amapá (IEPA).

Eight tick nymphs were collected from the skin of their hosts. The ticks were removed using tweezers and preserved in 70% alcohol. They were subsequently sent to the Laboratory of Zoology Collections at the Butantan Institute in São Paulo (IBSP) for morphological and molecular identification. The nymphs were identified based on the keys provided by Martins et al. (2010).

After morphological identification, all eight tick nymphs were sent for DNA extraction, which was carried out using a commercial kit (QIAGEN DNeasy Blood & Tissue Kit), following the manufacturer’s protocols. It is worth noting that, in the LCZ laboratory, all specimens were individually extracted and underwent a non-destructive extraction process. Prior to the extraction protocol, the ticks were punctured with a 30G insulin needle in the posterior region of the idiosoma. This procedure allows the internal contents to leak into the microtube, thereby increasing access to genetic material. Therefore, all specimens are preserved in the Acarological Collection under the supervision of the Collection Curator (co-author of the present study). The molecular characterization was performed targeting the 16S rRNA gene, using the following primer pairs: 16S+1 (5'-CTGCTCAATGATTTTTTAAATTGCTGTGG-3') and 16S-1 (5'-CCGGTCTGAACTCAGATCAAGT-3'). PCR reagent concentrations and thermal cycler conditions followed those of the original studies (Mangold et al., 1998). A negative control (type I ultrapure water; Invitrogen^®^) and a positive control (*Tyrophagus* sp. *pool*) were used for each reaction.

All the positive products were purified with ExoSap-IT (GE Healthcare® Pittsburgh, PA). Sanger sequencing was carried out at the Human Genome and Stem Cell Research Center of the Biosciences Institute of the University of São Paulo, in the state of São Paulo. The sequences thus obtained were assembled with Sequencing Analysis 5.3.1 and subjected to BLAST analysis (Altschul et al., 1990) to infer similarities with other sequences available in GenBank. Different genotypes were visually differentiated after an alignment using the CLUSTAL W algorithm (Thompson et al., 1994) implemented in Geneious R11

An aliquot of the extracted DNA was used in the molecular tests of the pathogens, following the bacterial and protozoan agents, target gene, and their original protocol: family Anaplasmataceae (16S rRNA) (Inokuma et al., 2000); *Bartonella* spp. (*nuoG*) (Colborn et al., 2010); *Coxiella* spp. (ITS region) (Hoover et al., 1992); *Hepatozoon* spp. (18S rRNA) (Spolidorio et al., 2009); *Mycoplasma* spp. (16S rRNA) (Maggi et al., 2013); and *Rickettsia* spp. (*gltA*) (Labruna et al., 2004) (Table 1).

**Table 1:** Primers and PCR conditions used in the detection of microorganisms in *A. ovale* collected from *P. cuvieri* specimens.

**Microorganisms**	***Primers* Sequences**	**PCR conditions**	**References**
Anaplasmataceae	5’-GGTACCYACAGAAGAAGTCC-3’	95 °C for 5’; 95 °C for 30”; 55 °C for 30”; 72 °C for 1,30’ (34x); 72 °C for 5’; 4 °C for ∞	Inokuma et al. (2000)
	5’-TAGCACTCATCGTTTACAG-3’		
*Bartonella* spp.	5’- GGCGTGATTGTTCTCGTTA-3’	94 °C for 5’; 94 °C for 30”; 53 °C for 30”; 72 °C for 1’ (35x); 72 °C for 5’; 4 °C for ∞	Colborn et al. (2010)
	5’- CACGACCACGGCTATCAAT-3’		
*Coxiella* spp.	5’- TATGTATCCACCGTAGCCAGTC -3’	95 °C for 5**’;** 95 °C for 30”; 60 °C for 30”; 72 °C for 1’ (40x); 72 °C for 7’; 4 °C for ∞	Hoover et al. (1992)
	5’- CCCAACAACACCTCCTTATTC -3’		
*Hepatozoon* spp.	5’- CGCGAAATTACCCAATTCTA-3’	95 °C for 3’; 95 °C for 15”; 53 °C for 40”; 72 °C for 40’ (40x); 72 °C for 5’; 4 °C for ∞	Spolidorio et al. (2009)
	5’- TAAGGTGCTGAAGGAGTCGTTTAT-3’		
*Mycoplasma* spp.	5′ - AGAGTTTGATCCTGGCTCAG - 3'	95 °C for 5’; 95 °C for 30”; 57 °C for 30”; 72 °C for 1’ (35X); 72 °C for 10’; 4 °C for ∞	Di Cataldo et al. (2020)
	5′ - TACCTTGTTACGACTTAACT - 3′		
	5′- GYATGCMTAAYACATGCAAGTCGARCG -3′		
	5′ - CTCCACCACTTGTTCAGGTCCCCGTC - 3′		
*Rickettsia* spp.	5′ - GCAAGTATCGGTGAGGATGTAAT- 3′	95 °C for 3**’;** 95 °C for 15”; 48 °C for 30”; 72 °C for 30” (40x); 72 °C for 7’; 4 °C for ∞	Labruna et al. (2004)
	5′ - GCTTCCTTAAAATTCAATAAATCAGGAT- 3′		

The obtained tick sequence was aligned with other homologous sequences retrieved from the GenBank database using the MAFFT software (Katoh & Standley, 2013) and edited via Geneious R11. W-IQ-Tree software (Trifinopoulos et al., 2016) was used to select the best evolutionary model, following the Bayesian information criterion (BIC), to construct phylogenetic analyses with the Maximum Likelihood method. Clade support was evaluated through 1,000 bootstrap replicates. The phylogenetic trees were edited using Treegraph 2.0.56-381 beta software (Stöver & Müller, 2010). Sequences of the species *Amblyomma aureolatum* were used as outgroups.

## Results

All the ticks were morphologically and molecularly identified as *Amblyomma ovale* and were deposited in the IBSP Collection under the numbers IBSP19180 and IBSP19181. The partial 16S rRNA sequences obtained from the eight nymphs corresponded to a single haplotype (GenBank accession number: PQ118967), which was 99.28% (query cover: 92%; e-value: 0.0) identical to *A. ovale* (MH513285) from French Guiana available in GenBank database. Therefore, this study expands the list of known hosts for *A. ovale.* PCR assays for pathogen detection did not show positive results for target genes.

**Figure 2:**
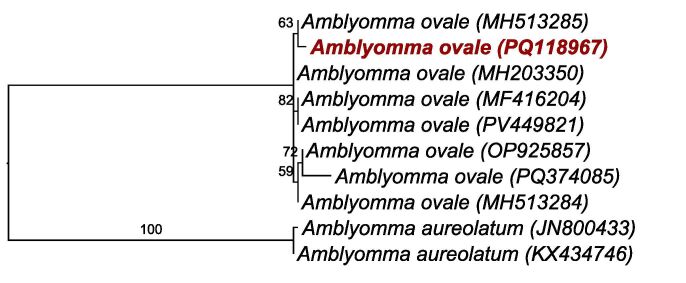
Phylogenetic analysis of the 16S rRNA sequences. An approximately 416-bp alignment was generated with HKY+I evolutionary model by Maximum Likelihood analysis. *Amblyomma aureolatum* (Ixodida: Ixodidae) were used as outgroup.

A Maximum Likelihood phylogenetic analysis was performed based on the 416 bp alignment of the 16S rRNA gene implemented with the HKY+I evolutionary model (Figure 2). The phylogenetic analysis positioned our sample in the same clade as other *A. ovale* ticks, proving the molecular identification of the tick collected.

## Discussion

*Amblyomma ovale*, the main hard tick species associated with small mammals of the families Cricetidae and Echimyidae, has been identified in studies in Brazil (Sponchiado et al., 2015). This study, which revealed the significantly expanded distribution area of this species, is the first report for *P. cuvieri.* Before this study, *A. ovale* was reported infesting the following Echimyidae species: *Proechimys canicollis, Proechimys guyannensis, Proechimys semispinosus, Proechimys quadruplicatus, Thrichomys apereoides, Thrichomys fosteri, Trinomys setosus* and *Euryzygomatomys spinosus* (Guglielmone et al., 2021). However, despite its wide geographical distribution and current scientific knowledge about this species, there are scant parasitological studies of this group. Therefore, this paper offers an unprecedented record of a parasitic association between *A. ovale* and *P. cuvieri*.

*Amblyomma ovale* has been associated with various mammalian hosts; its adult stage develops mostly on animals of the order Carnivora. However, rodents and other small warm-blooded vertebrates are recorded as hosts in their immature stages. In this context, our record corroborates the findings of Guglielmone et al. (2014) and Guglielmone et al. (2021). In view of the ability of *A. ovale* to associate with different mammals during its development phases, studies are needed to detect this tick, given its proven importance in the transmission of pathogens of the genus *Rickettsia* (Silva-Ramos et al., 2021).

Our study detected no pathogens in *A. ovale*, but this tick has previously been recorded in other regions naturally infected with *R. parkeri* strain Atlantic rainforest, which is responsible for pathological signs of spotted fever (Silva-Ramos et al., 2021). Under experimental conditions, these ticks have also proved to be competent in the transmission of *Hepatozoon canis* (Rubini et al., 2009).

Therefore, studies focusing on the detection of pathogens in these ticks in the Amazon region are not only strategic but also urgent. The variability of environments in which their possible hosts can move or settle favors contact with species of domesticated animals in and around human dwellings, potentially increasing human exposure to these ticks, and through them, to potential vectors of zoonotic diseases. This potential risk to human health underscores the need for action and surveillance in this area.

## Conclusions

The present study provides a new Neotropical Echimyidae host for *A. ovale*, contributing to the knowledge about the known hosts for ticks in the Amazon biome.

Although this study did not detect novel or previously documented pathogens, the newly identified association between *A. ovale* and *P. cuvieri* suggests that many undetected relationships between ticks and small mammals may still exist, which could encourage further pathogen screening in other unidentified associations.
